# Composite Hydrogel with Oleic Acid-Grafted Mesoporous Silica Nanoparticles for Enhanced Topical Delivery of Doxorubicin

**DOI:** 10.3390/gels10060356

**Published:** 2024-05-22

**Authors:** Marta Slavkova, Diana Dimitrova, Christina Voycheva, Teodora Popova, Ivanka Spassova, Daniela Kovacheva, Yordan Yordanov, Virginia Tzankova, Borislav Tzankov

**Affiliations:** 1 Department of Pharmaceutical Technology and Biopharmacy, Faculty of Pharmacy, Medical University-Sofia, 1000 Sofia, Bulgariahvoycheva@pharmfac.mu-sofia.bg (C.V.); tpopova@pharmfac.mu-sofia.bg (T.P.); btzankov@pharmfac.mu-sofia.bg (B.T.); 2 Institute of General and Inorganic Chemistry, Bulgarian Academy of Sciences, 1113 Sofia, Bulgaria; ispasova@svr.igic.bas.bg (I.S.); didka@svr.igic.bas.bg (D.K.); 3 Department of Pharmacology, Pharmacotherapy and Toxicology, Faculty of Pharmacy, Medical University-Sofia, 1000 Sofia, Bulgaria; yyordanov@pharmfac.mu-sofia.bg (Y.Y.); vtzankova@pharmfac.mu-sofia.bg (V.T.)

**Keywords:** doxorubicin, skin cancer, oleic acid grafting, amino-functionalized mesoporous silica, composite hydrogel

## Abstract

Mesoporous silica nanoparticles (MSNs) are inorganic nanocarriers presenting versatile properties and the possibility to deliver drug molecules via different routes of application. Their modification with lipids could diminish the burst release profile for water-soluble molecules. In the case of oleic acid (OA) as a lipid component, an improvement in skin penetration can be expected. Therefore, in the present study, aminopropyl-functionalized MSNs were modified with oleic acid through carbodiimide chemistry and were subsequently incorporated into a semisolid hydrogel for dermal delivery. Doxorubicin served as a model drug. The FT-IR and XRD analysis as well as the ninhydrin reaction showed the successful preparation of the proposed nanocarrier with a uniform particle size (352–449 nm) and negative zeta potential. Transmission electron microscopy was applied to evaluate any possible changes in morphology. High encapsulation efficiency (97.6 ± 1.8%) was achieved together with a sustained release profile over 48 h. The composite hydrogels containing the OA-modified nanoparticles were characterized by excellent physiochemical properties (pH of 6.9; occlusion factor of 53.9; spreadability of factor 2.87 and viscosity of 1486 Pa·s) for dermal application. The in vitro permeation study showed 2.35 fold improvement compared with the hydrogel containing free drug. In vitro cell studies showed that loading in OA-modified nanoparticles significantly improved doxorubicin’s cytotoxic effects toward epidermoid carcinoma cells (A431). All of the results suggest that the prepared composite hydrogel has potential for dermal delivery of doxorubicin in the treatment of skin cancer.

## 1. Introduction

The nanotechnological approach has been widely exploited for the improvement of drug delivery in various routes of administration. Nanoparticles can provide an opportunity for enhanced solubility and stability as well as targeted and modified drug release in order to reinforce the therapeutic effect. There is a vast number of nanocarriers which have been studied, including polymeric, inorganic and lipid nanoparticles and dendrimers. Each of them is characterized by specific advantages and disadvantages, and they have been further investigated to mitigate their limitations [1]. Mesoporous silica nanoparticles (MSNs) belong to the group of inorganic nanocarriers and have been a subject of considerable scientific research in recent years due to their versatile and tunable properties, which can lead to various drug release characteristics [2,3]. MSNs are outlined with a uniform pore size (2–50 nm), high pore volume, large surface area, biocompatibility and biodegradability [4]. Their application through the dermal route has been considered a new course for MSN investigation [5].

The surface of MSNs is evenly covered with silanol groups (Si-O-H) which can easily take part in a functionalization process. Co-condensation or grafting techniques can be applied to attach different moieties on the outermost and the nanopores’ surface [6]. These modifications could be utilized either to improve drug loading or achieve desirable release characteristics (modified, targeted, stimuli-sensitive, etc.). Covalently introducing amino groups to MSNs leads to enhanced potential for the loading of different chemotherapeutics [7,8,9,10,11]. Additionally, the amino groups could serve as an anchor for conjugation or interaction with different targeting or capping ligands [12].

Lipids present an interesting coating or grafting material, especially for the dermal route of administration, due to their biocompatibility and similarity to the lipid composition of the stratum corneum [13,14]. Silica nanoparticles coated with stearic acid in the presence of phosphatidylcholine showed improved skin permeation in comparison with non-coated nanoparticles [15]. In more recent studies, Pei et al. [16] and Zhu et al. [17] proposed the preparation of oleic acid-grafted mesoporous silica nanoparticles for parenteral and oral applications, respectively. Such a lipid layer can provide the nanoparticles with sufficient lipophilicity to prevent bursting and premature drug release [18]. Furthermore, the presence of oleic acid can contribute to improved membrane permeability of the nanocarrier [19] and enhance skin permeation due to disruption of the stratum corneum integrity [20]. Oleic acid is a monounsaturated omega-9 fatty acid present in many animal or vegetable oils [21]. It is biodegradable and non-toxic, and it is commonly used as a penetration enhancer in conventional and nanostructured dermal formulations [22,23]. Oleic acid-modified MSNs can be easily introduced into a suitable semisolid formulation for skin cancer therapy. Hydrogels represent one of the most appropriate vehicles for dermal delivery due to their ease of preparation, biocompatibility, patients’ compliance and ease of application and removal from the skin, as well as the possibility to bear different nanocarriers [24].

Doxorubicin (D) was selected as a model drug in the current study due to its water solubility [25]. It belongs to anthracycline antibiotics and possesses a pleiotropic effect against malignancies, including leukemia and solid tumors [26]. Furthermore, a significant number of publications showed its applicability in the treatment of different forms of skin cancer [27,28,29,30]. After systemic application, doxorubicin is distributed non-specifically and therefore may cause undesirable toxic effects in healthy tissues [31,32]. Its lack of specificity, the need of high doses and low concentration at the cancer site are prerequisite for toxicity issues such as myelosuppression, cardiotoxicity and alopecia [33]. Hence, topical delivery might be an approach for reduction of the drug’s side effects [27]. Improvement in the local concentration by actively applying a topical formulation could be a useful strategy to overcome the toxicity issues. However, doxorubicin’s hydrophilic properties render skin permeation negligible, which can limit its anticancer efficacy [34]. There are also data suggesting the development of multidrug resistance in melanoma cells due to doxorubicin’s active efflux through P-glycoproteins, thus resulting in therapeutic failure [27,35]. All of these characteristics are prerequisites for the need to improve its topical delivery by means of nanotechnology.

The application of oleic acid-grafted MSNs for dermal delivery has not been investigated thus far but could be a promising approach to enhancing its chemotherapeutic potential for dermal conditions. Therefore, the present work aims to investigate a relatively convenient approach for the treatment of skin malignancies. In this regard, amino-functionalized mesoporous silica nanoparticles are modified with oleic acid and subsequently loaded with doxorubicin. Their topical application is evaluated in terms of incorporating nanocarriers in a hydrogel formulation. The effectiveness of the proposed composite hydrogel is further evaluated in vitro on the epidermoid carcinoma cell line (A431).

## 2. Results and Discussion

### 2.1. Nanoparticle Grafting with Oleic Acid and Physicochemical Characterization

**Figure 1:**
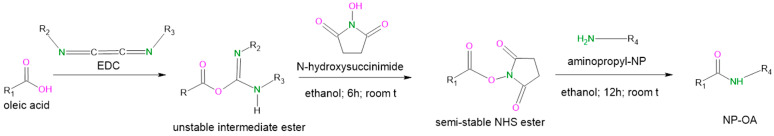
Mechanism of EDC/NHS reaction for preparation of NP-OA nanoparticles (modified from [36]).

Oleic acid (OA) was covalently bound to the parent aminopropyl-functionalized mesoporous silica nanoparticles via a carbodiimide/N-hydroxysuccinimide (EDC/NHS) reaction in ethanol. The mechanism of the synthesis reaction is shown in Figure 1.

The direct formation of amide groups is quite difficult without activation of the carboxylic group. One of the most suitable activation procedures is the one with EDC and NHS due to its simple procedure and high efficiency. The parent nanoparticles (NPs) used in the current study are aminopropyl-functionalized ones that possess terminal primary amino groups. Therefore, activated oleic acid can interact with those groups resulting in the chemically OA-modified aminofunctionalized nanoparticles (NP-OAs). The amount of available primary amino groups of the initial NPs was determined with a ninhydrin reaction and estimated to be 2.43 ± 0.3 wt%. After their engagement in an amide group, their amount dropped approximately in half (1.43 ± 0.4 wt%). Therefore, the newly obtained NP-OAs were grafted with 1.0 ± 0.4 wt% OA.

### 2.2. FTIR

**Figure 2:**
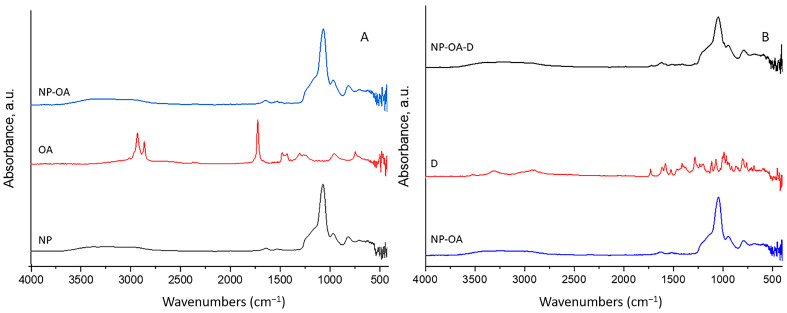
FTIR spectra of (**A**) NP, OA and NP-OA and (**B**) NP-OA, D and NP-OA-D.

Fourier transform infrared spectroscopy (FTIR) was used to prove the grafting of OA and the subsequent doxorubicin (D) loading. Figure 2 shows the FTIR spectra of the initial components and the prepared nanocarriers.

The peaks at 1049 cm^−1^ and 946 cm^−1^ in the spectrum of NPs were assigned to Si-O-Si asymmetric and symmetric stretching vibration of the Si-O-Si bond, respectively. The peak that appeared at 462 cm^−1^ is related to the bending vibration peak of the Si-O-Si bond [37]. The peaks of NP at 1627 cm^−1^ correspond to the bending vibration of silanol -OH [38]. The absorption bands near 1513 cm^−1^ and 682 cm^−1^ correspond to –NH_2_ symmetric and N-H bending vibration, respectively [39]. C-H stretches can be clearly identified at 2932 cm^−1^ and 2855 cm^−1^ in the NP sample. These results confirm the existence of an aminopropylsilane functional group in the parent NP sample. The FTIR spectrum of oleic acid showed two sharp peaks at 2853 and 2922 cm^−1^, corresponding to the symmetric and asymmetric -CH_2_ stretches, respectively [40,41,42,43]. The sharp peak observed at 1707 cm^−1^ was mainly due to asymmetric -C=O stretching, and the band at 1284 cm^−1^ corresponds to the C-O stretch of the carboxylic group. The peak at 1457 cm^−1^ reflects in-plane stretching of O-H, and that at 934 cm^−1^ reflects of out-of-plane OH stretching [40,41]. The FTIR spectrum of NP-OA showed a peak (-NH_2_) displaced at 1503 cm^−1^ due to the formation of an amide band as a result of the successful grafting of OA on the surface of the initial nanoparticles. Silanol groups were not engaged because there was no significant peak shift at 1049 cm^−1^, 945 cm^−1^ or 1627 cm^−1^. In the FTIR spectrum of doxorubicin, the bend peaks of the –NH_2_ groups were observed at 1615 cm^−1^ and 3528 cm^−1^. The stretch peak of the phenol ring was observed at 2900 cm^−1^, and the C=O stretching peak was observed at 1730 cm^−1^. The bend peaks of –C–N and –C–H were observed at 1379 and 869 cm^−1^, respectively. In the spectrum of loaded, oleic acid-grafted nanoparticles (NP-OA-D), the appearance of a new peak at 1720 cm^−1^ from C=O stretching validates the presence of D. The shift with 10 cm^−1^ compared with free D was most probably due to the H bonds with oleic acid, which confirms the broad peak at 3250 cm^−1^ in the spectrum of NP-OA-D. The pick of silanol groups in the spectrum of NP-OA-D was shifted to 1061 cm^−1^, which confirms their engagement with doxorubicin, probably due to H bonds. This gives us reason to consider that D was charged both in the NP pores and on the OA-functionalized surface.

### 2.3. Nitrogen Physisorption

**Figure 3:**
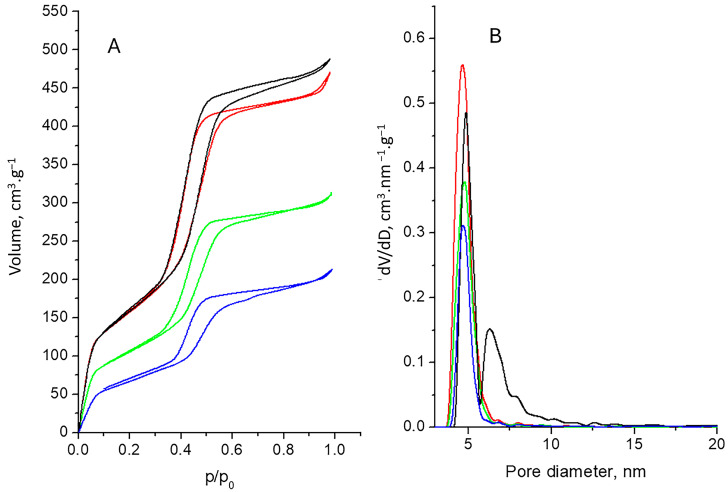
Adsorption-desorption isotherms (**A**) and pore size distributions (**B**) of NP (black line), NP-OA (red line), NP-D (green line) and NP-OA-D (blue line).

The adsorption-desorption isotherms, pore size distributions and values of the specific surface areas (S_BET_), total pore volumes (V_t_) and average pore diameters (D_av_) are shown in Figure 3 and Table 1.

The adsorption-desorption isotherms for all presented samples (Figure 3A) were of type IV, according to IUPAC classification [44], with a IVa isotherm final saturation plateau with an almost equal length, which is typical for mesoporous materials. This type is determined by the interaction between the adsorbent and adsorbate and because of the capillary condensation. In this case, hysteresis loops appeared to be attributed to H1 and are indicative of the availability of narrow-range mesopores, which are characteristic of the ordered structure of the material. The same was confirmed by the pore size distributions of the samples (Figure 3B). The uniform distribution for all samples, except for the NPs, where some bi-dispersion could be seen, is evident. Multimodal size distribution was observed in [45]. After OA grafting and D loading, all pores appeared to be in a rather narrow range, evidencing the well-organized mesoporous structure of the materials with no substantial change compared with the initial NPs. The texture parameters in Table 1 show that parts of the pores for the grafted NP-OA were filled, thus slightly decreasing the specific surface area and the total pore volume. After doxorubicin loading, the specific surface areas and the total pore volumes of NP-D and NP-OA-D decreased significantly but without distortion of the ordered structure. However, relative preservation of average pore diameters of about 5 nm revealed some redistribution of the porous space. In [17,46], the authors pointed out that the drug loading capacity for mesoporous silica nanoparticles with pore diameters of more than 5 nm was higher than that on such particles with pores 2.3 nm in diameter. Similar adsorption isotherms, hysteresis loops, pore size distributions and texture parameters were reported in [45,47,48] for silica nanoparticles, while in [18], the authors presented isotherms and texture parameters indicating practically filled mesopores, significant pore blocking and the subsequent absence of detectable mesoporosity.

### 2.4. X-ray Diffraction

**Figure 4:**
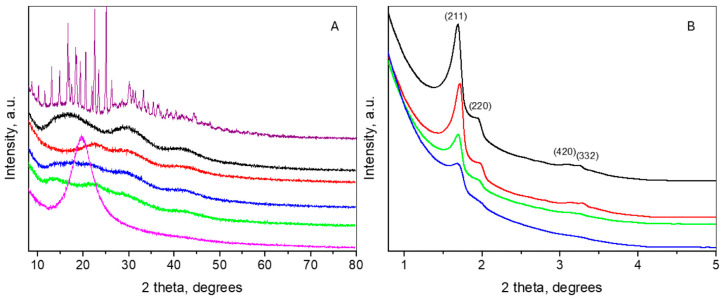
Wide-angle (**A**) and small-angle (**B**) XRD patterns of doxorubicin (purple line), oleic acid (magenta line), NP (black line), NP-OA (red line), NP-D (green line) and NP-OA-D (blue line).

The wide-angle part (see Figure 4A) of the diffraction pattern of doxorubicin represents a large number of peaks, corresponding to the high crystallinity of the phase. The mean crystallite size was determined to be 83 nm. Indexing of the reflections was performed within Space Group *P2_1_*, following the structure description given in [49]. The determined unit cell parameters were a = 10.241(2) Å, b = 6.224(1) Å, c = 20.307(4) Å and beta = 96.65(1)°, which were quite close to those reported in [49]. The wide-angle part of the NP pattern revealed an amorphous state with three broad humps at about 17°, 30°, and 42°2θ, which differed from the pattern of MCM-48, displaying only one amorphous peak at around 22.5° [50], probably due to the aminopropyl functionalization. The pattern of air-dried oleic acid was characterized by only one intensive peak at 19.5°2θ. OA grafting onto the NPs resulted in shifting of the first peak of NP to 22°2θ, while the positions of the other NP peaks remained unchanged. On the other hand, doxorubicin loading on NP was expressed in the appearance of a new peak positioned at roughly 14°2θ, suggesting amorphization of the drug. The pattern of NP-OA-D may be considered a superposition of the patterns of NP-OA and NP-D. The changes in the number and positions of the peaks in the wide-angle XRD patterns are indicative of the successful loading of oleic acid and doxorubicin.

The small-angle parts of the patterns of NP, NP-OA, NP-D and NP-OA-D are presented in Figure 4B. The appearance of peaks in this range is indicative of the ordered mesoporous structure of the mentioned samples. All diffraction patterns were similar and could be indexed in Space Group Ia-3d, being related to a cubic, three-dimensional pore structure such as that of MCM-48 [51]. The determined unit cell parameters of the cubic cells for NP, NP-OA, NP-D and NP-OA-D were 125(1) Å, 123(1) Å, 121(1) Å and 121(1) Å, respectively. The intensities of the peaks for the grafted and loaded samples were lower than that of NP, and the lowest intensity was observed for the NP-OA-D sample. This fact implies partial pore filling, which confirms the findings from nitrogen physisorption and the literature data [46,52].

### 2.5. Drug Loading and Encapsulation Efficiency

Doxorubicin was successfully loaded onto both the parent (NP-D) and OA-grafted nanoparticles (NP-OA-D), as was shown by the FTIR and XRD analysis. The data regarding the encapsulation efficiency showed that propylamine-functionalized mesoporous nanoparticles can almost encapsulate completely the drug (Table 2). This is in accordance with the literature [7], suggesting improved doxorubicin loading. The high efficiency of drug loading in the current study could be explained by the modified method for drug loading. The optimal ratios between the drug and aminopropyl-functionalized MSNs was based on a study by He et al. [7], but the impregnation was carried out until complete evaporation of the solvent, rather than for 24 h. Furthermore, the NPs in the present study were aminopropyl-functionalized and possessed a similar surface area but larger pore volume and pore size than the cited reference. As shown by the physisorption and XRPD data, doxorubicin was loaded mostly within the pores, and therefore almost complete encapsulation efficiency was observed. A similar high loading efficiency and capacity of about 19% were reported in other studies [7,11,53,54]. In the case of NP-OA-D, surface outer loading was observed (as suggested by the FTIR data), which resulted in a slightly lower efficiency. The cubic pore structure can also be the reason for the observed values.

### 2.6. Particle Size, Polydisperisty and Zeta Potential

**Figure 5:**
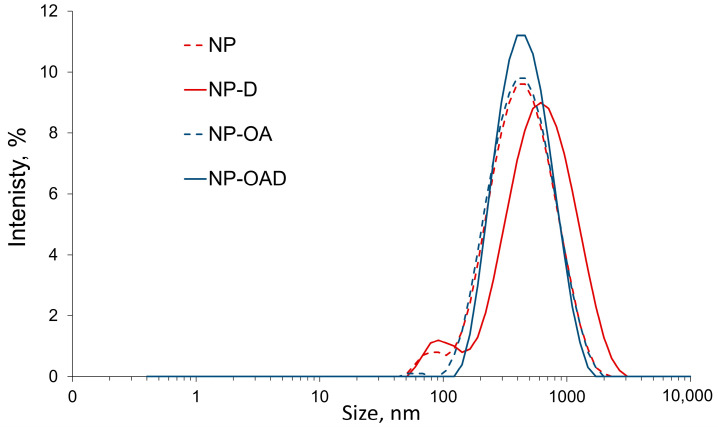
Z-average diameter of the nanoparticles measured with DLS.

The results of the dynamic light scattering (DLS) analysis are presented in Table 2. The different hydrodynamic diameters of the tested samples are shown on Figure 5. There was a limited increase in the sizes as a result of the doxorubicin loading or OA grafting. All nanoparticles were with narrow particle sizes and a polydispersity index (PDI) between 0.142 and 0.359, suggesting appropriate preparation and loading procedures.

The observed negative zeta potential of the pristine NPs (Table 2) can be due to the predominant amount of free silanol groups on the MSNs’ surface (2.43 ± 0.3 wt% amino content, according to the ninhydrin reaction). Similar findings can be seen in the literature, suggesting that an amino content of less than 25% results in nanoparticles with a negative zeta potential in distilled water [55]. The zeta charge depends significantly on the amount of free amino groups on the MSNs’ surface and can range from negative (approximately −25 mV) to positive values (approximately +10 mV) [11,56]. The change in the zeta potential and particle size (Table 2) shows that oleic acid was successfully attached to the mesoporous silica nanoparticles. It engaged approximately half of the amino groups (1.0 ± 0.4 wt% oleic acid, according to the ninhydrin reaction), and thus only silanol groups were responsible for the observed zeta charge and its absolute value increases. The loading of doxorubicin (NP-D) led to a further increase in the absolute value of the zeta potential. This finding is in accordance with previous studies [11].

The loading of doxorubicin after oleic acid conjugation resulted in a decreased absolute value of the zeta potential. This was most likely due to the interaction of the free silanol groups with doxorubicin molecules and their engagement in hydrogen bonding. Similar findings have been demonstrated by other researchers [57]. After OA is conjugated, the available amino groups are reduced and more difficult to reach for the drug molecules. Therefore, predominantly hydrogen bonding between doxorubicin and the free silanol groups can be expected.

### 2.7. TEM Analysis

**Figure 6:**
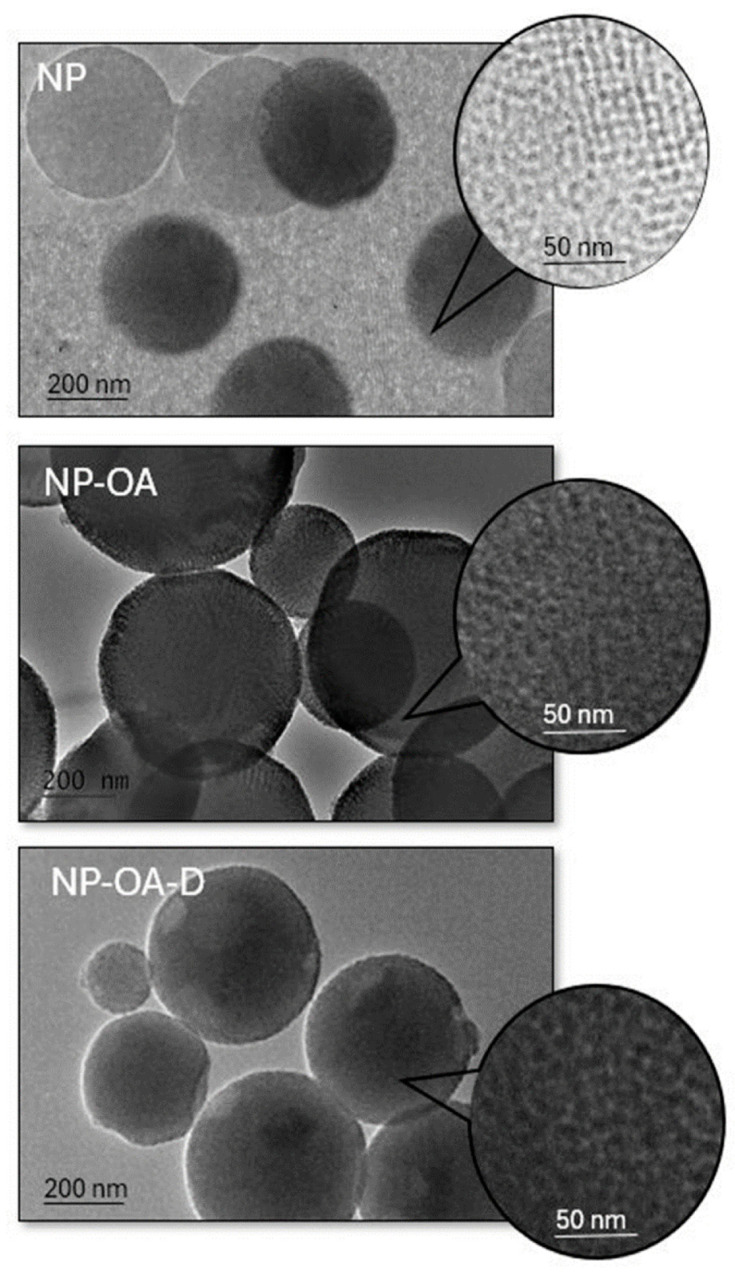
TEM micrographs at different magnifications: 25,000× (**left**) and 100,000× (**right**).

TEM studies were performed to evaluate the structure of the parent MSNs and to investigate possible changes upon modification and drug loading. The obtained micrographs are presented on Figure 6. The porous structure of the NPs is clearly visible. The attachment of OA led to partial obscuring of the pores, and a halo around the particles’ surface was observed. The doxorubicin loading clearly led to filling of the pores. The results are in accordance with the DLS and N_2_ physisorption analysis.

### 2.8. In Vitro Dissolution Test

**Figure 7:**
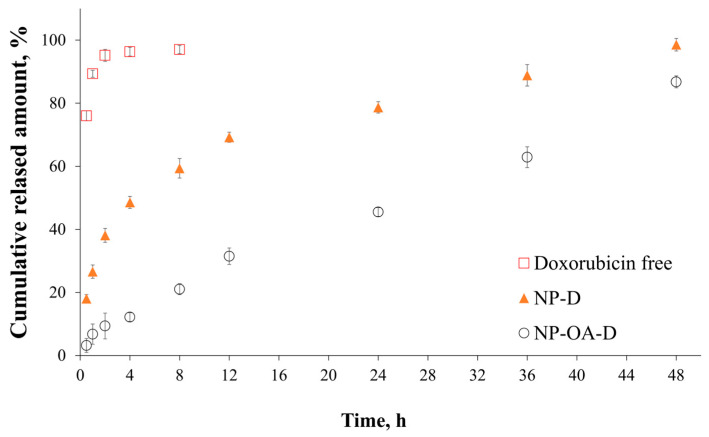
In vitro release profiles for free doxorubicin, NP-D and NP-OA-D.

The in vitro release studies (Figure 7) show that free doxorubicin dissolved rapidly in the aqueous medium at 32 °C. Opposed to this, the doxorubicin loading onto the nanocarriers was associated with delayed release following different release mechanisms, as shown in the kinetics studies (Table 3). Releasing processes from mesoporous systems occurred after solvent penetration into the meso-channels followed by the drug’s dissolution and diffusion, extending the release rate. Similar results for amino-functionalized silica loaded with doxorubicin can be found in the literature [7,58].

The oleic acid grafting led to a decrease in the burst release, and approximately 86% of the doxorubicin was released within 48 h. Similar data have been shown for other drugs in OA-modified mesoporous nanoparticles [17]. Functionalization with the lipid reduces the hydrophilicity of the carrier, and therefore, in aqueous medium, a delayed release is observed.

In addition, the release kinetics of the prepared nanoparticles were investigated. The results of the fitting are presented in Table 3. The release kinetics from the parent nanoparticles followed the Kormsmeyer–Peppas model, as the correlation coefficient (R^2^) was greater than the ones in the other models. The release exponent was *n* ≤ 0.43, which suggests that Fickian diffusion was the main mechanism of drug release. This is in accordance with previous studies [59]. In the case of modification with oleic acid, the release followed a first-order (R^2^ = 0.989) or Higuchi model (R^2^ = 0.984). To the best of our knowledge, the release kinetics from oleic acid-modified MCM-48 nanoparticles were not investigated. It is evident that the release is dependent on the doxorubicin concentration and governed by diffusion. Such assumptions were made by other studies with other modifications of mesoporous nanoparticles with water-soluble drugs [50,60,61].

### 2.9. Physicochemical Characterization of the Hydrogels

Different hydrogels were prepared in order to compare the effects of various parameters on their properties. The concentration chosen was based on the typical concentration established for its application as a single gelling agent [62,63]. All gels showed optimal pH levels (in the range of 5.7–6.9) for dermal application, even though there were some differences upon the inclusion of nanoparticles and oleic acid, as shown in Table 4.

Upon application, the semisolid formulations could exert an occlusive effect. The gels loaded with lipid nanoparticles or lipids containing hybrid nanoparticles, such as the ones prepared in the current study, can also have an influence on water loss from the substrate onto which they are applied. This may affect the depth of permeation, as it changes the barrier function of the skin. Lipids are known to exert significant occlusiveness on the skin due to their lipophilic nature. Lipid nanocarriers have also shown a significant effect on water loss due to their large surface area [64,65]. The development of oleic acid-grafted MSNs may also affect the water loss and related skin hydration. It has been shown that there is a linear relationship between the in vitro occlusion and in vivo skin hydration, and at the same time, the latter promotes drug permeation [65]. The occlusive properties of the formulations were evaluated, and petrolatum was used as a positive control due to its known occlusive effects [66]. The spreadability (S_F_) and occlusion factor (F) for petrolatum were determined to be 7.35 ± 1.86 and 99.62 ± 0.14, respectively. It can be seen (Table 4) that the incorporation of nanoparticles within poloxamer gel (GNP-D and GNP-OA-D) caused a decrease in the observed occlusion compared with the gel containing free doxoubicin (GD). Even the OA-modified nanoparticles did not show significant alterations in the occlusive factor in comparison with the pristine nanoparticles loaded with doxorubicin. As a comparison, it can be seen that free OA in the same amount led to increased occlusive properties. This is probably related to the lipophilic nature of OA, which was maintained when it is incorporated as a free component into the gel.

Spreadability is an important quality for a prepared formulation, which is related to its extraction from the container and ease of application on the skin. The systems were evaluated at 25 °C, and the results are presented in Table 4. The gel containing free oleic acid (G+OA+D) had a decreased spreadability factor compared with the gel without it (GD). These results are in accordance with previously published reports. There are investigations showing that the presence of more than 5% oleic acid leads to significant alterations in the viscosity and thermosensitive properties of 25% poloxamer gel [67]. In the present work, the incorporation of 1% oleic acid did not change the gelling properties of the poloxamer significantly. Therefore, the decrease in the spreadability factor was less pronounced. The presence of free doxorubicin was accompanied by increased viscosity. This was probably due to its aqueous solubility, and thus the free water for the poloxamer was limited. Furthermore, the oleic acid was emulsified by the poloxamer, and this was probably the reason for the slight increase in viscosity and decreased spreadability. The incorporation of nanoparticles into the poloxamer gel led to a slight decrease in the spreadability factor, but it was not affected by the type of nanoparticle. A similar decrease in the spreadability upon the inclusion of nanoparticles within poloxamer 407 gels has been shown in the literature [68]. Based on the presented data in Table 4 (spreadability, G′ and G″), the gels containing nanoparticles were weaker than the gels containing free doxorubicin with and without OA. This suggests that the presence of MSNs interferes with gel formation. The mechanism of gelation with poloxamer takes place with the polymer self-assembling into nanosized micelles followed by their packing into a three-dimensional network [69]. In the literature, an interaction between different types of nanoparticles and poloxamer hydrogels has been reported [70,71].

**Figure 8:**
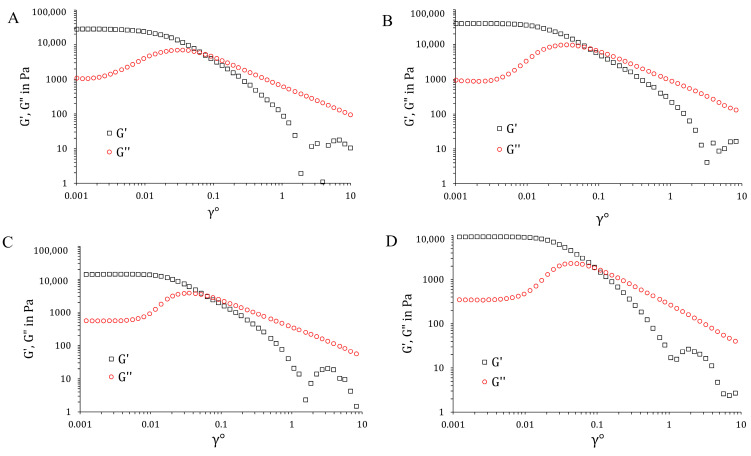
Oscillation amplitude sweep test with storage modulus (G′) and loss modulus (G″) as a function of strain amplitude γ_0_ with the percentage for poloxamer based gels at 32 ± 0.5 °C: (**A**) GD, (**B**) G+OA+D, (**C**) GNP-D and (**D**) GNP-OA-D.

An oscillation amplitude sweep test was used to determine a material’s linear visco-elastic range, where the stress and strain amplitudes had a linear relationship (Figure 8). The four samples exhibited similar behavior, which are typical for physical gels. The samples were characterized by a nearly constant G′ and G″ at rather small strain values, where G′ >> G″. In this region, the elastic component was dominant. Increasing the strain led to slight shear thickening, followed by pronounced shear thinning. In other words, at high shear stress, the material changed from highly elastic to viscous. The effect of shear thinning is associated with a breakup of the closely packed micellar structures and subsequent sliding of micellar layers in the flow direction. This is related to the mechanism of poloxamer gelation by micelle formation and the packing into a three-dimensional network structure thereafter [69].

Subsequently, the frequency sweep test was performed at a fixed stress value (0.002), which is in the linear viscoelastic range (determined by the oscillation amplitude sweep test). All samples behaved as hard gels; the moduli were independent of the applied frequency, and the elastic response to stresses greatly surpassed the viscous one (G′ >> G″). In addition, these two curves no longer crossed over in the investigated frequency range.

### 2.10. In Vitro Permeation Test

**Figure 9:**
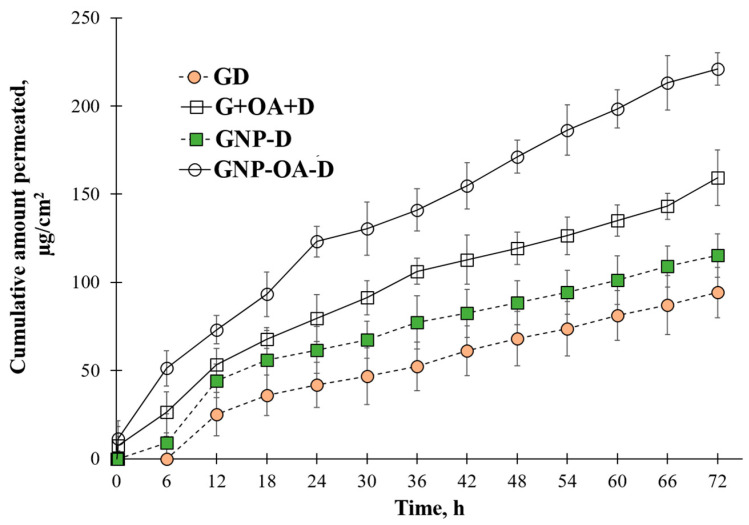
Cumulative amount of doxorubicin permeated in Franz diffusion cell in pH 5.5 at 32 °C, where mean ± SD and n = 3. GD = free doxorubicin in gel; G+OA+D = gel with free doxorubicin and oleic acid; GNP-D = nanoparticles loaded with doxorubicin in gel; GNP-OA-D = oleic acid-modified nanoparticles loaded with doxorubicin in gel.

The permeation profiles depicted in Figure 9 show the differences in the doxorubicin behavior for the different gel formulations. A delayed release was evident in all cases. The higher viscosity of the semisolid base slowed down the diffusion of the drug through the gel itself, and a lag time was observed. The profile of doxorubicin permeation from the gel loaded with free drug (GD) showed a limited permeated amount over 72 h. The parameters of the permeation study are shown in Table 4.

The addition of free oleic acid led to 1.69 fold improved permeation of doxorubicin, as was expected due to its known penetration-enhancing properties [22,23]. The limited effect of the free oleic acid (G+OA+D compared with GD) was probably due to a change in the physicochemical properties of the gels. Even though the viscosity increase was limited, the spreadability data show the presence of more stiff gel. This slowed down the diffusion of the dissolved drug through the semisolid base and counteracted the penetration-enhancing properties of OA to some extent. The nanoparticles (GNP-D) presented improved permeation profiles in comparison with the free drug (1.22 fold). This was most likely due to the hydrophilic nature of doxorubicin, which showed higher affinity toward the hydrogel and not the model membrane. The loading of the drug into the nanoparticles reduced its affinity to the semisolid base. In addition, the small particle size can facilitate skin permeation, as was shown by other researchers [72]. Furthermore, the gel viscosity was reduced in comparison with the free doxorubicin and OA-containing gels. Therefore, drug diffusion was hindered to a lesser extent, and the drug reached the acceptor medium faster. The gels with NP-OA-D showed additional improved permeation, namely 1.91 fold in comparison with the non-modified nanoparticles. It can be elaborated that a synergistic effect was observed between the nanoparticles and their surface modification with the penetration enhancer. The negative zeta potential observed for the nanoparticles is another possible explanation for the difference in the permeation of the drug through the model membrane [73]. When comparing the zeta potential between the NP-D and NP-OA-D (Table 1), it can be seen that the pristine nanoparticles with doxorubicin had a significantly higher absolute value for the zeta potential in comparison with the modified and D-loaded ones (−40 mV and −12 mV, respectively). The keratinocytes at the physiological conditions exhibited a negative charge. Therefore, the greater the negative potential of the nanoparticles, the higher the repulsion with the dermal cells will be. All of these observations contributed to the 2.35 fold improvement in permeation in comparison with the free drug-loaded gel. It can be concluded that the factors affecting the penetration are quite complex and interact with each other. Further studies may be necessary in order to elucidate the effect of each factor and their interaction.

The present results suggest that oleic acid attached to the surface of aminopropyl-functionalized mesoporous silica nanoparticles can promote better skin penetration. The low concentration of OA (1%) only led to modification of the nanocarriers’ affinity toward the membrane and improved diffusion through it. Higher concentrations have been shown to impede the release due to significant alteration of the lipophilicity of the formulation as well as its viscosity [67,74].

There are some limitations in the conducted experiment, as the membrane used resembles skin but is still a replacement membrane. Further studies would be needed to investigate the ex vivo and in vivo behavior of the formulations and evaluate skin retention in different layers. In addition, it is worth noting the difference in the malignant cells compared with the healthy ones, and therefore, the effect needs to be evaluated in actual conditions in the future.

### 2.11. Cell Experiments

#### 2.11.1. Cytotoxicity in A431 Cells

**Figure 10:**
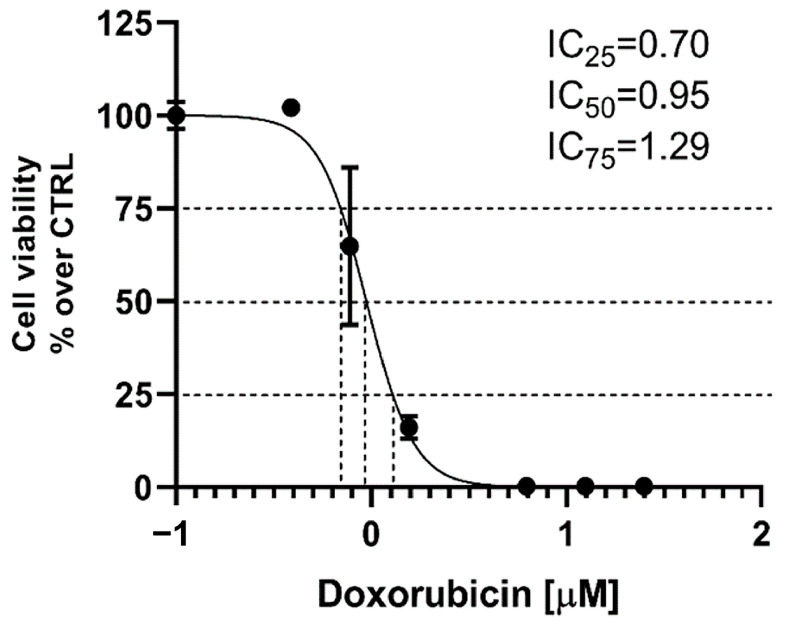
Concentration–response curve of epidermoid carcinoma A431 cells treated with D. The curve was fitted with nonlinear regression, and the IC25, IC50 and IC75 values were calculated.

The IC values for doxorubicin were calculated based on a preliminary cell viability assay on A431 cells treated with D (0.4 and 6.25 µM). The results showed that treatment with D resulted in a steep decrease in viability of the A431 cells, with IC25, IC50 and IC75 values of 0.70, 0.95 and 1.29 µM, respectively (Figure 10). These concentrations of D were chosen for further experiments.

**Figure 11:**
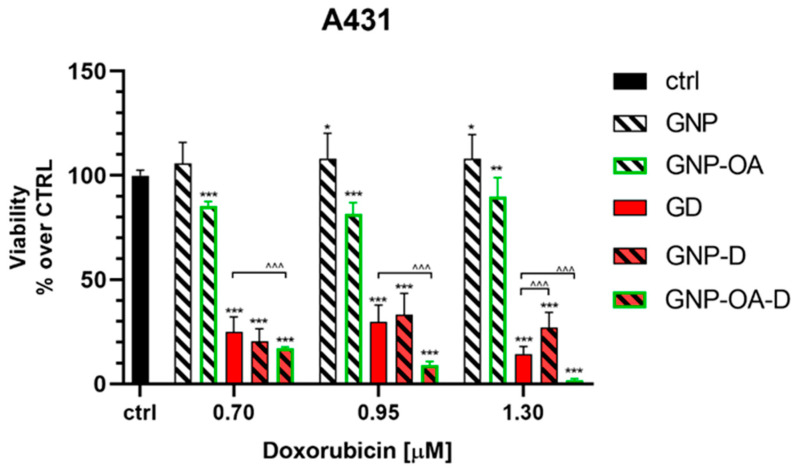
Cell viability of A431 cells treated for 72 h with non-loaded GNP and GNP-OA (6 mg/mL), free D (0.70 µM (IC25), 0.95 µM (IC50) or 1.29 µM (IC75)), GNP-D and GNP-OA-D. The GNP-D and GNP-OA-D dispersions contained the same D concentrations as the free D. Data are presented as mean ± SD. Statistics: one-way ANOVA with Dunnett’s post hoc test. * *p* < 0.05; ** *p* < 0.01 and *** *p* < 0.001 versus control. Multiple *t*-tests with Holm-Sidak correction, where ^^^ *p* < 0.001 versus D.

To evaluate the cytotoxicity effects of doxorubicin loaded in non-functionalized (GNP-D) and functionalized nanoparticles (GNP-OA-D) on epidermoid carcinoma cells, the A431 cells were treated for 72 h with either free or loaded D at concentrations of 0.70 µM, 0.95 µM or 1.30 µM (Figure 11). As can be seen, both the non-loaded GNP and GNP-OA were non-toxic, according ISO standard 10993-5 [75]. The loading of doxorubicin into the non-functionalized NPs (GNP-D) did not change the cytotoxicity significantly compared with the free non-loaded drug. It is noteworthy that loading of doxorubicin in functionalized GNP-OA-D resulted in a strong cytotoxic effect in the A431 cells, manifesting in a several-fold, dose-dependent increase in doxorubicin cytotoxicity. At a doxorubicin concentration of 1.30 µM, practically all A431 cells underwent cell death (about 2% viability). This corresponded to a greater than seven-fold decrease compared with the effect of free GD (14.3% cell viability) at the same concentration.

#### 2.11.2. Phase-Contrast Micrography of A431 Cells

**Figure 12:**
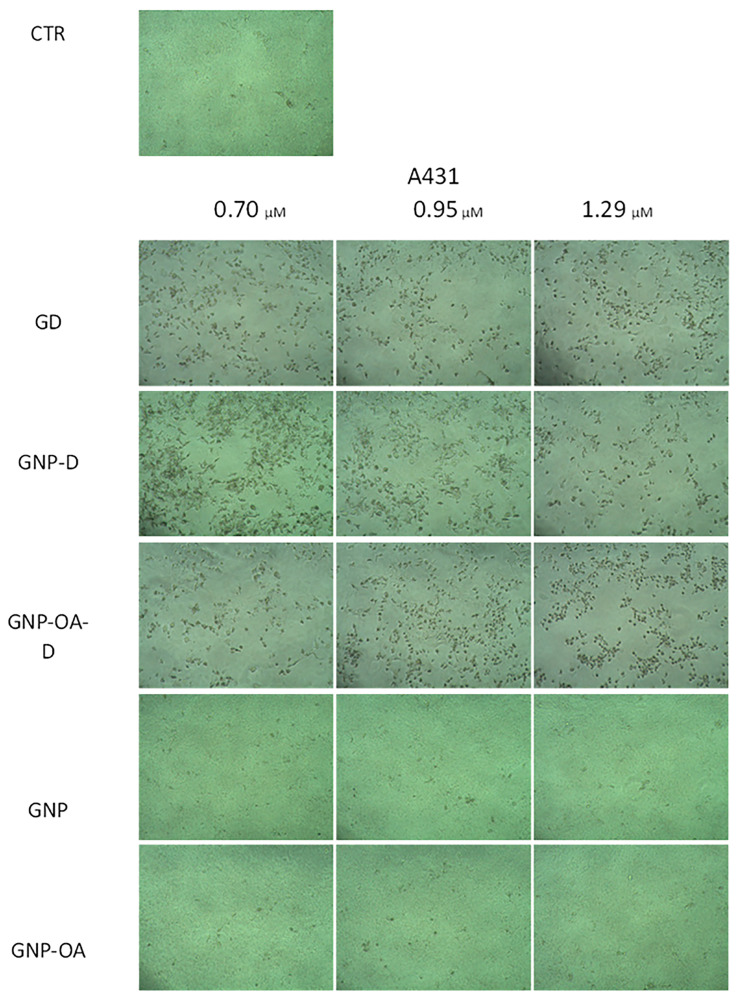
Phase-contrast micrographs of A431 cells treated for 72 h with non-loaded GNP and GNP-OA (6 mg/mL), free D (0.70 µM (IC25), 0.95 µM (IC50) or 1.29 µM (IC75)), GNP-D and GNP-OA-D (D concentrations corresponding to free D) (100× magnification).

Visual evaluation of phase-contrast micrographs of the A431 cells show that GNP and GNP-OA treatment did not result in significant visual changes in the cells compared with the control, except for a slight change in texture, probably due to the presence of gel (Figure 12). Unlike in the control group images, treatment with GD resulted in the appearance of visible detached circular cells, which is characteristic for apoptotic cells. The amount of rounded cells was slightly higher at 1.29 µM GD compared with the lower concentrations. The addition of GNP-D resulted in the appearance of not only apoptotic cells at 0.7 µM but also deformed cells, which seemed to be damaged but still not apoptotic. The increase in the GD concentrations (0.95 and 1.29 µM) resulted in the decrease of such deformed cells and the appearance of only rounded cells. However, treatment with functionalized, loaded GNP-OA-D did not cause the appearance of deformed cells after treatment with any of the applied concentrations and only yielded rounded cells.

Our study showed that the loading of doxorubicin in particles resulted in preservation of its in vitro antiproliferative effects, and functionalization of the drug-delivery system with OA resulted in a significant improvement.

The viability results were visually confirmed in phase-contrast micrographs, and the presence of rounded and detached cells after doxorubicin treatment could be an indication that a fraction of the cells was apoptotic [76]. Doxorubicin has been shown to exert both caspase-dependent and caspase-independent cell death in different cell lines [77]. Oleic acid has been shown to induce apoptosis in hepatocellular carcinoma cells, unlike normal hepatocytes [78]. The antiproliferative effects of OA have also been shown in experiments with other tumor cell lines, originating from low metastatic breast (MCF-7) and gastric (SGC7901) carcinomas [79]. The potential mechanisms by which oleic acid functionalization improves the cytotoxic effects of doxorubicin could be related to improved cellular uptake [80] and the propensity of OA to decrease the expression of oncogenes [81], which are responsible for A-431 cell survival, interfere with lipid metabolism and affect autophagy [82]. Chronic fatty acid intake has been shown to be tumorigenic in some occasions, and there should be careful benefit-risk assessment when specific therapeutic applications are designed [83,84].

## 3. Conclusions

The present study demonstrated successful attachment of oleic acid onto aminopropyl functionalized mesoporous silica nanoparticles. The prepared nanocarriers achieved high encapsulation efficiency (97.6 ± 1.8%) and a delayed release profile of doxorubicin. They were successfully incorporated into a hydrogel based on poloxamer 407, aiming for dermal application. The characteristics of the gel in terms of spreadability, pH, occlusion and viscosity were suitable for the intended purpose. The findings of the current study also showed improved in vitro permeation of the OA-modified nanoparticles in comparison with the free physical system and non-modified NPs. In vitro cell studies on A431 cells treated with the test substances showed that loading OA-modified nanoparticles is a promising strategy for improving doxorubicin’s cytotoxic effects toward epidermoid carcinoma cells. Therefore, it can be concluded that the proposed composite hydrogel could be a suitable drug delivery platform for doxorubicin in the treatment of skin cancer.

## 4. Materials and Methods

### 4.1. Materials

Doxorubicin hydrochloride (D), propylamine-functionalized silica (NP) with a 4 nm pore size, oleic acid (OA), N-ethyl-N′-(3-(dimethylamino)-propyl) carbodiimide (EDC), N-hydroxysuccinimde (NHS), 3-aminopropyltriethoxysilane (APTES) and petrolatum were purchased from Sigma Aldrich (Burlington, MA, USA). Pluronic F127 was obtained from BASF (Ludwigshafen, Germany), and ninhydrin was obtained from Merck (Darmstad, Germany). All other reagents were of analytical grade and used as obtained. Distilled water was prepared in the lab.

### 4.2. Nanoparticle Grafting with Oleic Acid

The OA was grafted onto the amino-functionalized silica following the procedure proposed by Pei et al. [16] with some modifications. The conjugation was based on chemical interaction between the –NH_2_ group of the MSN and the –COOH group of OA. Briefly, the carboxylic groups of OA (125 µL) were activated in an ethanol solution (5 mL) of EDC (100 mg) and NHS (110 mg). The mixture was magnetically stirred for 6 h at room temperature. Then, NPs (50 mg) were introduced into the mixture, and it was left for 12 more hours with gentle stirring in the dark. The grafted nanoparticles (NP-OA) were separated with centrifugation and washed subsequently with water and ethanol to remove any unreacted components. The particles were dried at room temperature and further characterized.

### 4.3. Ninhydrin Reaction

The amount of grafted OA molecules on the MSN surface was determined indirectly based on the decrease in the number of –NH_2_ groups. This was achieved with a ninhydrin reaction according to the procedure proposed by Lu [85]. A fresh solution of 3% ninhydrin in 0.5 M acetate buffer was prepared. The tested NP and NP-OA nanoparticles (0.5 mg) were dispersed and sonicated in 1 mL acetate buffer. The standard ninhydrin solution (2 mL) was added to the samples. A blank was prepared by mixing the ninhydrin solution with 1 mL acetate buffer. All of the prepared samples were heated at 100 °C for 15 min to form a colorful complex. Then, they were cooled to room temperature and centrifuged for 5 min at 6000 rpm. The supernatant was evaluated spectrophotometrically at λ = 570 nm. The amount of primary amines was calculated based on the absorbance, with a predetermined calibration curve of APTES as supposed by Sakeye and Smått [86].

### 4.4. Drug Loading and Encapsulation Efficiency

D (25 mg) was loaded by following the solvent impregnation method. The drug was dissolved in 4 mL of distilled water. The NP-OA nanoparticles (100 mg) were suspended afterward in the solution under magnetic stirring. The mixture was continuously stirred and protected from direct light until complete evaporation of the solvent. Then, the obtained NP-OA-D nanoparticles were washed three times with ethanol and water, dried at room temperature in the dark and further used for characterization.

The efficiency of doxorubicin encapsulation (*EE%)* was calculated based on the amount of doxorubicin included in the loading procedure (*D_TOTAL_*) and the amount found in the collected supernatant upon washing the obtained NP-OA-D (*D_FREE_*). For this purpose, the following equation was used:

(1) $$EE\%=\frac{D_{TOTAL}-D_{FREE}}{D_{TOTAL}}\times 100$$

In addition, the loading capacity (wt%) was calculated based on Equation (2) as proposed in [54]:

(2) $$LC\%=\frac{D_{TOTAL}-D_{FREE}}{{Weight}_{laoded\;NP}}\times 100$$

The analysis was performed at λ = 480 nm using a UV spectrophotometer (Thermo Scientific Evolution 300, Madison, WI, USA) and based on a previously obtained standard curve with known concentrations.

### 4.5. Fourier Transform Infrared Spectroscopy (FTIR)

A Thermo-Nicolet FTIR instrument equipped with an attenuated total reflectance (ATR) device (Thermo Scientific Nicolet, Waltham, MA, USA) was used to collect IR spectra in the range of 4000–400 cm^−1^ and with a resolution of 4 cm^−1^ for the parent, OA-grafted and doxorubicin loaded nanoparticles.

### 4.6. Nitrogen Physisorption

The textural characteristics of the samples were determined by low-temperature nitrogen physisorption in a static volumetric Quantachrome NOVA 1200e instrument (Anton Paar Quanta Tech Inc., Boynton Beach, FL, USA).

### 4.7. X-ray Diffraction

The powder X-ray diffraction analysis was performed with a Bruker D8 Advance diffractometer (Karlsruhe, Germany) with Cu Kα radiation using a Lynx Eye detector in the 5–80°2θ range. The small angle part of the patterns was collected using a manually adjustable knife edge in the range of 0.4–5°2θ with a step of 0.02°2θ.

### 4.8. Particle Size, Polydispersity Index and Zeta Potential

Dynamic light scattering analysis was used for the determination of the nanoparticles size, polydispersity index and zeta potential. This was performed with the help of Zeta Master (Zetasizer Nano ZS, Malvern Instruments, Worcestirshire, UK). The samples were prepared immediately before measuring. Different nanoparticles (NP, NP-D, NP-OA and NP-OA-D) were dispersed in distilled water, sonicated for 2 min, filtered through 0.45 µm Whatman cellulose filter and measured at 25 °C and a scattering angle of 90°. All of the measurements were performed in triplicate.

### 4.9. Transmission Electron Microscopy (TEM)

The sizes and pore structures of the NP, NP-OA and NP-OA-D samples were characterized with a transmission electron microscope (JEOL JEM 2100 h STEM (200 kV; point resolution = 0.23 nm), JEOL, Tokyo, Japan). Water dispersions of the corresponding nanoparticles were dropped on a polymer microgrid supported on a Cu grid. The water was then evaporated under a vacuum, and the samples were investigated at different magnifications.

### 4.10. In Vitro Drug Dissolution and Kinetics Study

The dissolution profiles of free doxorubicin, NP-D and NP-OA-D were investigated in 1 mL of distilled water at 32 ± 0.5 °C in an incubator shaker. All test samples corresponded to 1mg of doxorubicin. At predetermined time intervals, the samples were centrifuged, and the amount of drug in the supernatant was measured spectrophotometrically at λ = 480 nm. Fresh release medium was introduced, and the dissolution continued at the same conditions. The average cumulative release data of three experiments were used to study the release kinetics. The best fit was determined in the case of the correlation coefficient (R^2^) being closest to 1.

### 4.11. Hydrogel Preparation, Nanoparticles Incorporation, Appearance and pH

Pluronic F127 (25% *w*/*w*) was dissolved in water following the cold method; specifically, it was dispersed in distilled water and left to dissolve completely at 4 °C overnight. The obtained gel was further used to embed the nanoparticles by dispersion at about 10 °C. The amount of nanoparticles dispersed corresponded to a 2 mg/g gel concentration of doxorubicin, and they were called GNP-D and GNP-OA-D. Two other gels with free drug were prepared for comparison purposes. Doxorubicin was dissolved in the water, and Pluronic F127 was then added for gelation, resulting in GD formulation. A physical mixture of doxorubicin and oleic acid corresponding to the GNP-OA-D amounts was also incorporated into the same type of gel and was labeled G+OA+D.

The prepared hydrogels were visually evaluated for their appearance. The pH levels of the diluted gel samples (1% *w*/*w*) were measured potentiometrically with a pH meter (Hanna HI98100, Hanna Instruments Inc., Woonsocket, RI, USA).

### 4.12. Hydrogel Rheology and Spreadability

Dynamic rheological measurements were conducted in controlled deformation (CD) mode with a parallel plate sensor system (top plate diameter = 20 mm; gap = 1 mm). Three runs of each sample were performed at 32 °C. The oscillation amplitude sweep tests were carried out at a frequency of 1 Hz in a ɣ_0_ range from 0.001 to 10. The frequency sweep tests were carried out in the 0.2–10 Hz frequency range at ɣ_0_ = 0.002.

A modified parallel plate method was used for evaluation of the gel’s spreadability [87]. All of the gels were kept at room temperature for 24 h prior to investigation. Briefly, a gel sample (approximately 1 g) was placed carefully on a glass plate located on the surface of a desktop scanner (HP Scanjet G3110). Another glass plate with a known weight was placed on top. Then, every 1 min, a new weight (up to 600 g) was added. An image was taken with the help of the scanner before the new weight was positioned. The surface of the spread gel was calculated afterward using Image J software (version 1.49q, National Institute of Health, Bethesda, MD, USA). The spreadability factor (*S_F_*) (in mm^2^/g) was calculated based on the following equation:

(3) $$S_{F}=\frac{A}{W}$$

where *A* (mm^2^) is the maximal area of spread calculated and *W* (g) is the total weight added. The averaged spreadability factor was calculated for each formulation as a mean of all individual *S_F_*. All experiments were performed in triplicate.

### 4.13. In Vitro Occlusion Test

In the present research, the methodology for water evaporation proposed by Caldas et al. was adapted with some modifications [88]. The tested gels (1 g) were evenly spread (spread area of 13.84 cm^2^) on a Whatman cellulose filter (0.45 µm) previously placed on a 50 mL glass beaker. As a reference, one of the samples did not have gel applied on the filter. The beakers were filled with 25 mL of distilled water, covered with the filter and sealed with Teflon tape. Each of the as-prepared systems was precisely weighed and then kept at a constant temperature of 32 ± 0.5 °C and relative humidity of 50% ± 1% in the dark for 48 h. At predetermined time intervals, the samples were weighed to assess water loss. The occlusive factor (*F*) was used to compare the samples. It was calculated based on the water loss in the reference (*L_R_*) and the water loss of the gel samples (*L_S_*) according to the following equation:

(4) $$F=\frac{L_{R}-L_{S}}{L_{R}}\times 100$$

An occlusive factor of *F* = 0 equals no occlusive effect, and *F* = 100 represents maximal occlusion. The petrolatum sample was used as a positive control for the occlusion experiment [66].

### 4.14. In Vitro Permeation Study

The permeation of the prepared gel formulations was evaluated in vitro in accordance with the EMA guideline on topical formulations [89]. The apparatus used was a Logan system 913-6 automated transdermal diffusion cell sampling system (Logan Instruments Corp., 19C Schoolhouse, Somerset, NJ, USA). The test was carried out according to the procedure proposed by Kazmi et al. and Saleem and Idris [90,91] with some alterations. As a membrane to separate the donor and accepting compartment, an egg membrane was used [90,91,92]. The egg shell membrane was prepared as described by Shah et al. [92]. It was carefully extracted and checked under a microscope for any damage. The membrane was attached between the donor and receptor compartments of the Franz diffusion cell, with an effective surface area of 1.54 cm^2^. The investigated gels were equilibrated at room temperature for 6 h prior to the test. Samples corresponding to approximately 1 mg of doxorubicin were introduced into the donor compartment. As an acceptor medium, phosphate buffer with a pH of 5.5 and volume of 12 mL was applied. The permeation study was conducted at 32 ± 0.5 °C with continuous stirring over 72 h. At predetermined time intervals, aliquots were withdrawn and replaced with fresh medium. The amount of drug permeated was assessed spectrophotometrically at λ = 480 nm. All samples were tested in triplicate. The cumulative amount of the drug that penetrated through the unit membrane surface (*Q_T_*, µg/cm^2^) was calculated based on the following equation [72,93]:

(5) $$Q_{T}=\frac{C_{n}\cdot V_{A}+\sum_{i}^{n-1}C_{i}\cdot V_{s}}{A}$$

where *Q_T_* is the cumulative amount of doxorubicin which penetrated through the unit membrane surface; *C_n_* is the doxorubicin concentration in the *n*th sample; *C_i_* is the doxorubicin concentration in the *i*th sample; *V_A_* is the volume of the acceptor phase (12 mL); vs. is the volume of the sample (1 mL) and *A* is the effective permeation area of the membrane (1.54 cm^2^). Furthermore, the data of cumulative amount *Q_T_* was plotted versus time, and the steady state flux (J_ss_, µg/cm^2^/h) was calculated from the slope in the linear part of the plotted curve [72,93].

### 4.15. Cell Experiments

#### 4.15.1. Cell Culture

The epidermoid carcinoma (A431) cell line was obtained from The European Collection of Authenticated Cell Cultures (ECACC, Salisbury, UK). A431 cells were cultured in Dulbecco’s Modified Eagle’s Medium (DMEM) (high glucose, supplemented with 10% FBS and L-glutamine) in an incubator, maintaining 37 °C, 5% CO_2_ and maximum humidity.

#### 4.15.2. Cytotoxicity Assay

The cells were plated in 96 well plates at suspension densities of 1 × 10^4^ cells/well and allowed to attach overnight. The cells were then treated with doxorubicin (D) or doxorubicin, loaded into nanoparticles (GNP-D) and either not functionalized or functionalized with oleic acid (GNP-OA-D). All of the substances were introduced to the cell culture medium as concentrated hydrogel dispersions in a sol state. The non-loaded GNP and GNP-OA were introduced at a concentration of 6 mg/mL, which were shown to be non-toxic in preliminary experiments. The D concentrations used in the experiments were based on the IC25, IC50 and IC75 values obtained from a preliminary experiment with A431 cells (D concentrations: 0.1–25 µM). After 72 h of treatment, the liquid component of each well was aspirated and exchanged with fresh medium containing 3-(4,5-dimethylthiazol-2-yl)-2,5-diphenyltetrazolium bromide (MTT) solution [94]. After 2 h of incubation, the wells were aspirated again, and their contents were exchanged with dimethylsulfoxide (DMSO). After 30 min of incubation, absorbance was measured at 570 nm with a reference wavelength of 690 nm with a plate reader (Synergy 2, BioTek Instruments, Inc., Highland Park, Winooski, VT, USA).

#### 4.15.3. Phase Contrast Micrography

Immediately before the MTT assay, micrographs of each treatment group and control group were taken with an 8 MP CCD digital camera (Optikam Pro 8LT-4083.18LT) in an Optika XDS-2 inverted phase-contrast microscope (OPTIKA Srl, Ponteranica, Italy).

#### 4.15.4. Statistical Analysis

All statistical analyses were performed with GraphPad Prism version 8.0.0 for Windows (GraphPad Software, San Diego, CA, USA). IC25, IC50 and IC75 were interpolated from a nonlinear regression model. The treatment groups were compared by means of one-way ANOVA with Dunnett’s post hoc test (versus controls). D-loaded GNP and GNP-OA were compared to D by means of multiple *t*-tests with Holm-Sidak correction for multiple testing.

## Figures and Tables

**Table 1:** Texture parameters.

Sample	S_BET_ (m^2^·g^−1^)	V_t_ (m^2^·g^−1^)	D_av_ (nm)
NP	586	0.76	5.3
NP-OA	576	0.74	5.2
NP-D	387	0.48	5.0
NP-OA-D	250	0.33	5.1

**Table 2:** Nanoparticle coding, dynamic light scattering analysis data and encapsulation efficiency.

Sample Coding	Full Name	Size (nm) (Mean ± SD)	PDI	Zeta Potential (mV) (Mean ± SD)	EE (%) (Mean ± SD)	LC (%) (Mean ± SD)
NP	Aminopropyl-functionalized silica	352.7 ± 2.6	0.251	−23.9 ± 0.3	-	-
NP-D	NP loaded with D	449.3 ± 3.1	0.359	−40.7 ± 0.2	99.14 ± 0.83	19.63 ± 0.09
NP-OA	OA grafted NP	372.4 ± 2.9	0.219	−50.2 ± 0.4	-	-
NP-OA-D	NP-OA loaded with D	402.8 ± 3.3	0.142	−14.5 ± 0.4	97.66 ± 1.81	18.96 ± 0.23

**Table 3:** Kinetic parameters of the in vitro drug release from the parent and OA-modified nanoparticles.

Formulation	Zero Order	First Order	Higuchi	Korsmeyer–Peppas
	$Q_{t}=Q_{0}-k_{0}t$	$lnQ_{t}=lnQ_{0}-k_{1}t$	$Q_{t}=k_{H}t^{1/2}$	$\frac{M_{t}}{M_{\infty }}=k\cdot t^{n}$
NP-D	R^2^ = 0.803	R^2^ = 0.9249	R^2^ = 0.9435	R^2^ = 0.983
	k = 2.366	k = −0.024	k = 14.421	n = 0.312
NP-OA-D	R^2^ = 0.970	R^2^ = 0.989	R^2^ = 0.984	R^2^ = 0.908
	k = 1.785	k = −0.011	k = 10.103	n = 0.762

*Q* = amount of drug; *k* = rate constant; *t* = time; *n* = release exponent.

**Table 4:** Physicochemical and permeation parameters of the prepared hydrogels (n = 3).

Gel Formulations with	Physicochemical Property	Mean	SD
Free D (GD)	pH	5.75	0.21
	F, (%) (after 48 h)	57.19	0.52
	S_F_ (mm^2^/g)	3.20	0.74
	ŋ (Pa·s)	4521	121
	G′ (Pa)	28,390	149
	G″ (Pa)	1032	67
	J_ss_ (µg/cm^2^/h)	1.12	0.35
	Q_t_ (µg/cm^2^)	94.23	11.56
NP-D (GNP-D)	pH	6.01	0.23
	F (%) (after 48 h)	53.12	0.73
	S_F_ (mm^2^/g)	2.70	0.74
	ŋ (Pa·s)	2161	98
	G′ (Pa)	13,570	203
	G″ (Pa)	388.4	97
	J_ss_ (µg/cm^2^/h)	1.34	0.31
	Q_t_ (µg/cm^2^)	115.33	15.36
Free D and OA (G+OA+D)	pH	5.15	0.09
	F (%) (after 48 h)	62.81	0.76
	S_F_ (mm^2^/g)	2.87	0.42
	ŋ (Pa·s)	4573	103
	G′ (Pa)	28,720	91
	G″ (Pa)	819.1	86
	J_ss_ (µg/cm^2^/h)	1.93	0.27
	Q_t_ (µg/cm^2^)	159.33	17.25
NP-OA-D (GNP-OA-D)	pH	6.91	0.17
	F (%) (after 48 h)	53.91	0.47
	S_F_ (mm^2^/g)	2.87	0.42
	ŋ (Pa·s)	1486	162
	G′ (Pa)	9327	174
	G″ (Pa)	363.2	73
	J_ss_ (µg/cm^2^/h)	2.72	0.12
	Q_t_ (µg/cm^2^)	221.01	16.81

G = gel; D = doxorubicin; OA = oleic acid; NP = nanoparticle; F = occlusion factor; S_F_ = spreadability factor; ŋ = dynamic viscosity; G′ = elastic modulus; G″ = loss modulus; J_ss_ = permeation flux; Q_t_ = cumulative penetrated amount.

## Data Availability

The data are contained within the article.
